# Assessment of cardiotoxicity and plasma ropivacaine concentrations after serratus intercostal fascial plane block in an experimental model

**DOI:** 10.1038/s41598-022-26557-5

**Published:** 2023-01-02

**Authors:** Matilde Zaballos, Olalla Varela, Ignacio Fernández, Lucía Rodríguez, Sergio García, Oscar Quintela, Elena Vázquez, María-José Anadón, Jesús Almendral

**Affiliations:** 1 Department of Forensic Medicine, Psychiatry and Pathology, Complutense University, Madrid, Spain; 2 Department of Anesthesiology, Hospital General Universitario Gregorio Marañón, C/ Dr Esquerdo nº46, 28007 Madrid, Spain; 3 Hospital General Universitario Gregorio Marañón, Madrid, Spain; 4 Hospital Monteprincipe, Grupo HM Hospitales, University CEU-San Pablo, Madrid, Spain

**Keywords:** Cardiology, Medical research, Risk factors

## Abstract

Serratus intercostal fascial plane block (SIFPB) has emerged as an alternative to paravertebral block in breast surgery. It involves the administration of high volumes and doses of local anesthetics (LA) that can potentially reach toxic levels. Ropivacaine is widely used in thoraco-fascial blocks; however, there is no information on the plasma concentrations attained after SIPFB and whether they are associated with cardiotoxicity. Plasma concentrations of ropivacaine and its electrophysiological effects were evaluated in eight pigs after bilateral SIFPB with ropivacaine in doses of 3 mg/kg. Plasma concentrations, electrophysiological and hemodynamic parameters were measured sequentially for the following 180 min until the end of the study. The area under the curve, the maximum plasma concentration (C_max_) and the time to reach C_max_ (t_max_) were calculated. The median arterial ropivacaine concentration C_max_ was, 2.34 [1.40 to 3.74] µg/ml. The time to reach the highest concentration was 15 [10 to 20] min. Twenty-five percent of the animals had arterial concentrations above the lower limit concentration of ropivacaine for LA systemic toxicity (3.4 µg/ml). No alterations were observed in the electrophysiological or electrocardiographic parameters except for a prolongation of the QTc interval, from 489 ± 30 to 544 ± 44 ms (Δ11.38 ± 6%), P = 0.01. Hemodynamic parameters remained in the physiological range throughout the study. SIFPB with ropivacaine in doses of 3 mg/kg has reached potentially toxic levels, however, it has not been associated with adverse electrophysiological or hemodynamic effects.

## Introduction

Breast cancer is one of the most common malignancies among women and surgical resection of the primary tumor with axillary dissection remains the cornerstone therapy of patients with breast cancer^1^. The anesthetic strategy usually consists of the combination of general anesthesia supplemented with local or regional anesthesia with the aim of optimizing acute pain control and patient comfort^2^.

Ultrasound-guided fascial plane blocks have generated great interest as an alternative analgesic technique to the thoracic paravertebral block (PVB), considered the gold standard analgesic modality in breast surgery^2–6^. The interfascial plane between the serratus anterior and intercostal muscles contains the branches of the intercostal nerves and their blockade at the mid-axillary line would provide analgesia in the region of the breast and axilla. The block is termed serratus intercostal fascial plane block (SIFPB) or (BRILMA) that is, blockade of the branches of the intercostal nerves in the mid-axillary line ^6,7^.

Perioperative breast pain control with fascial plane blocks comprises the administration of a relatively large volume of local anesthetic (LA), usually 25–30 ml of aminoamide LA in a single dose^7–9^. The anatomical region of the SIFPB is widely vascularized and, like other regional techniques, potentially toxic plasma concentrations of LA can be reached ^8–10^.

Ropivacaine, a long-acting LA, has been widely used for regional anesthesia, including several fascial plane blocks. However, there are no data on its absorption kinetics and plasma concentrations after SIFPB block and we have no information on whether the ropivacaine concentration achieved is associated with any signs of cardiotoxicity.

Our aim was to quantify plasma concentrations of ropivacaine after ultrasound- guided SIFPB and to assess whether these plasma concentrations of ropivacaine are associated with relevant cardiotoxicity in a porcine experimental model.

We hypothesize that ropivacaine in doses of 3 mg/kg does not lead to cardiotoxic plasma concentrations after ultrasound-guided SIFPB in a porcine experimental model.

## Materials and methods

### Ethics

Ethical approval for this study (Ethical Committee N° PROEX 260/16) was provided by the Ethical Committee of Animal Studies of Hospital General Universitario Gregorio Marañon, Madrid, Spain and the Environment and Animal Protection department of Madrid (Chairperson Jesús Carpintero Hervás) on 22 November 2016.

All procedures were carried out in accordance with the principles for the care and treatment of experimental animals and followed the national and local recommendations of the Spanish Ministry of Agriculture. The study lasted from January 2018 to December 2018. The study is reported in accordance with ARRIVE guidelines.

### Animals

Eight healthy mini pigs of either sex, 5 females and 3 males, with a median weight of 36.1 kg [33 to 38] were used in our experimental protocol. They were kept fasting for 12 h before the experiments, with free access to water.

### Interventions

The animals were sedated with ketamine (20 mg/kg)^11^ intramuscularly and a caudal auricular vein was subsequently cannulated. Anesthesia was induced with intravenous thiopental at a dose of 5–10 mg/kg and was maintained with sevoflurane at 1 minimal alveolar concentration (MAC) for pigs (2.66%)^12^.

Pigs were intubated and controlled ventilation was started at a tidal volume of 6–8 ml/kg, with a respiratory rate of 12 breaths per min (Heinen & Löwenstein Leon respiratory ventilator; Direx SL, Wiesbaden, Rheinland-Pfalz, Germany). The respiratory parameters were adjusted to maintain an arterial partial pressure of carbon dioxide between 35 and 40 mmHg. Throughout the experiment an infusion of 0.9% sodium chloride was administered at a rate of 5 ml kg/h. Esophageal temperature was maintained Esophageal temperature was maintained between 37.5 and 39 °C with a forced-air warming blanket (Warm Touch™ 5300A, Mallinckrodt Medical SA, Madrid, Spain).

**Figure 1:**
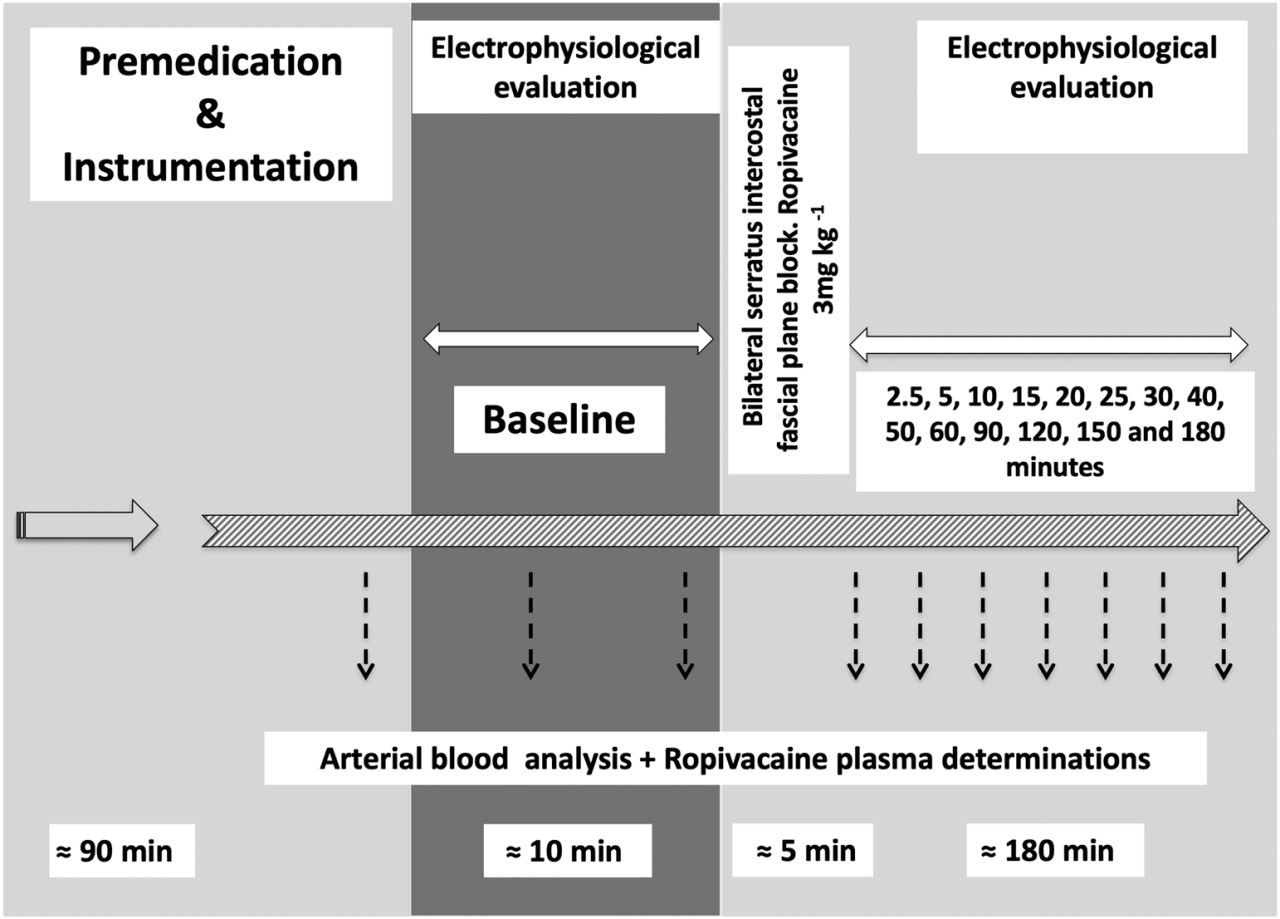
Experimental protocol. Eight animals were anesthetized and instrumented. After a period of stabilization, a baseline electrophysiological assessment was performed followed by a bilateral serratus intercostal fascial plane block with ropivacaine in doses of 3 mg/kg. Plasma concentrations, electrophysiological and hemodynamic parameters were measured sequentially for the following 180 min until the end of the study.

Animal instrumentation was as previously described^13–15^. We used a closed-chest porcine experimental model that allowed reliable cardiac electrophysiological evaluation of cardiotoxicity of local anesthetic agents (Fig. 1).

The femoral vessels (veins and arteries) were cannulated percutaneously by ultrasound (Vivid S5; GE Healthcare, Wauwatosa, WI). A 5-French catheter (PiCCO, Pulsion Medical Systems AG, Munich, Germany) was inserted through the femoral artery and used to record continuous hemodynamic parameters and for cardiac output (CO) measurement by transpulmonary thermodilution. The contralateral femoral artery was cannulated for arterial blood gas sampling and analysis of plasma ropivacaine concentration.

Three quadripolar catheters—one of them deflectable (Marinr, Medtronic, Minneapolis, MN), were inserted via the femoral veins under fluoroscopic guidance. The catheters were positioned in the right atrium, right ventricle, and in across the His bundle area. Catheters were used for stimulation and intracardiac electrogram recordings, which were high-pass (70 Hz) and low-pass (500 Hz) filtered. His bundle recordings were obtained with special care to avoid recording right bundle branch potentials. Stimulation was accomplished with a customized programmable stimulator (CS3 Cardio-stimulator; A.S.P. Electronic Medical, Madrid, Spain).

Electrocardiographic parameters and intracavitary electrograms were continuously monitored, recorded and stored on a computer-based digital amplifier/recorder system. (LABSYSTEM PRO EP Recording System; Boston Scientific Corporation, Marlborough, MA).

The animals were allowed to stabilize for 15 min before initiating the experimental protocol.

### Serratus intercostal fascial plane block

All blocks were performed by a single person (O.V.) with knowledge and training in ultrasound-guided regional techniques and experience in performing SIFPB on pigs ^14,15^. A GE 6–12 MHz linear array probe was used, and the blocks were performed with the animals positioned in supine. A two-plane ultrasound scan was performed to identify the serratus anterior muscle, and the fifth and sixth ribs in the mid-axillary line. The SIFPB was performed with a echogenic needle (50 mm Stimuplex^®^ Ultra 360, B. Braun Medical S.A. Barcelona, Spain) introduced by an in-plane approach and after confirmation by hydrodissection, 15 ml of ropivacaine was administered on each side, visualizing the spread of the local anesthetic solution. Each animal received a total dose of 3 mg/kg ropivacaine.

### Measurements

The electrophysiological parameters measured were as follows: sinus cycle length (SCL), heart rate, PR interval, QRS duration, AH interval (the time from the earliest rapid deflection of the atrial electrogram in the His bundle recording to the onset of the His deflection), HV interval (the time from the beginning of the His bundle deflection to the earliest onset of ventricular activation), QT and corrected QTc intervals (using the Bazett formula [QTc = QT/√R − R]).

Since local anesthetics slow ventricular conduction in a frequency-dependent manner as a result of blockade of cardiac sodium channels^16^, this effect was evaluated by measuring the QRS duration after ventricular pacing trains of 10–12 beat with interstimulus intervals of 400 or 500 ms (stQRS_400_ or stQRS_500_). To facilitate a precise QRS duration measurement during rapid ventricular stimulation, the QRS onset was timed to the pacing spike during right ventricular pacing and the QRS offset was timed to the latest lead (considering all 12 leads). The duration of the last QRS complex of each paced train was measured. Pacing was bipolar (5 mm interelectrode distance) at an intensity of 10 mA and with a pulse width of 1 ms.

In all groups, the electrophysiological parameters were measured at baseline and at predetermined intervals: (2, 5, 10, 15, 20, 30, 40, 50, 60, 90, 120, 150 and 180 min after completion of bilateral SIFPB).

The hemodynamic parameters were measured at baseline and at predetermined intervals: (10, 30, 60, 90, 150 and 180 min after completing bilateral SIFPB).

Arterial blood gas measurements were performed throughout the study, but for statistical evaluation, baseline values and data at 60, 120 and 180 min after SIFP block were included.

### Ropivacaine quantification

Ropivacaine concentrations in arterial and venous plasma were assessed at baseline and at predetermined intervals (2.5, 5, 10, 15, 20, 25, 30, 40, 50, 60, 90, 120, 150 and 180 min after completion of bilateral SIFPB).

The concentration of ropivacaine was assessed by liquid chromatography coupled to tandem mass spectrometry/mass spectrometry using the method described previously for the analysis of bupivacaine, which was modified to determine the concentrations of ropivacaine^13,14^.

### Outcomes

The primary outcome was to quantify arterial and venous plasma ropivacaine concentrations after SIFPB and to analyze the time course of these concentrations over 180 min.

The secondary outcome was to analyze whether the plasma concentrations of ropivacaine reached after SIFPB were associated with cardiac electrophysiological toxicity effects.

### Statistical analysis

#### Sample size

For sample size estimation, the reported plasma arterial concentration of ropivacaine 4.3 ± 0.6 µg/ml with potential toxicity were considered^17^, compared with transversus abdominis plane (TAP) block as a typical fascial block. For TAP blocks, the mean maximum arterial plasma concentrations of ropivacaine have been reported as 1.56 ± 0.5 µg/ml^17^. Accepting an alpha risk of 0.05 and a beta risk of 0.2 in a bilateral contrast, 7 were required to detect a difference equal to or greater than 0.8 µg/ml, based on previous studies. A standard deviation of 0.7 µg/ml was assumed. Finally, 8 animals were included to replace possible losses.

#### Data analysis

The analysis was conducted using the SPSS statistical package (version 20.0; SPSS Inc., Chicago, IL, USA). Results are presented as medians and and interquartile range (IQR) for non-normally distributed data using the Shapiro–Wilk test, and as means ± standard deviation for normally distributed variables. Ropivacaine plasma concentrations were represented by the area under the curve (AUC, trapezoid method). The maximum plasma concentration (C_max_) and the time to reach C_max_ (t_max_) were calculated.

Repeated measures analysis of variance was used to analyze the evolution of the electrophysiologic and hemodynamic parameters over time.

Comparisons between baseline electrophysiological parameters and those obtained during peak ropivacaine concentration were performed with paired Student’s t-test.

### Euthanasia

At the end of the experiment, the pigs were given a bolus of propofol and potassium chloride for euthanasia.

### Conference presentation

This study has been presented in the 2018 World Congress on Regional Anesthesia & Pain Medicine, ASRA’s 43rd Annual regional Anesthesiology & Pain Medicine Meeting.

## Results

All the animals completed the study, which lasted 299 ± 18 min. Biological parameters are shown in Table 1. They all remained stable and in physiological range throughout the study.

**Table 1:** Biologic parameters at baseline, 60, 120 and 180 post-serratus intercostal fascial plane block.

	Baseline	60 min-SIFPB	120 min-SIFPB	180 min-SIFPB	P value
Na^+^ (mmol/l)	138 (137–40)	139 (137–140)	138 (136–140)	138 (137–140)	0.98
K^+^ (mmol/l)	3.5 (3.3–3.7)	3.7 (3.6–4)	3.7 (3.6–3.9)	3.8 (3.7–3.9)	0.11
Ca^++^ (mmol/l)	1.3 (1.28–1.34)	1.3 (1.27–1.37)	1.37 (1.3–1.41)	1.37 (1.32–1.4)	0.006
pH	7.53 ± 0.02	7.52 ± 0.04	7.53 ± 0.03	7.54 ± 0.02	0.43
PaO_2_ (mmHg)	458 ± 54	474 ± 86	481 ± 76	478 ± 73	0.53
PaCO2 (mmHg)	39 ± 3.9	38 ± 2.5	38 ± 3	38 ± 3	0.51
HCO_3−_ (mmol/l)	33.3 ± 4.3	31.7 ± 3	31.8 ± 3.3	32.5 ± 3.4	0.10
EB (mmol/l)	10.3 ± 4.2	8.7 ± 3.2	8.8 ± 3.4	9.4 ± 3.3	0.33
SaO2 (%)	100	100	100	100	1
Hematocrit (%)	25 (23–26)	24 (23–25)	25 (22–26)	25 (23–26)	0.95

Data are showed as medians interquartile range (IQR) for non-normally distributed data using the Shapiro–Wilk test, and as means ± standard deviation for normally distributed variables.

*SIFPB* serratus intercostal fascial plane block.

**Figure 2:**
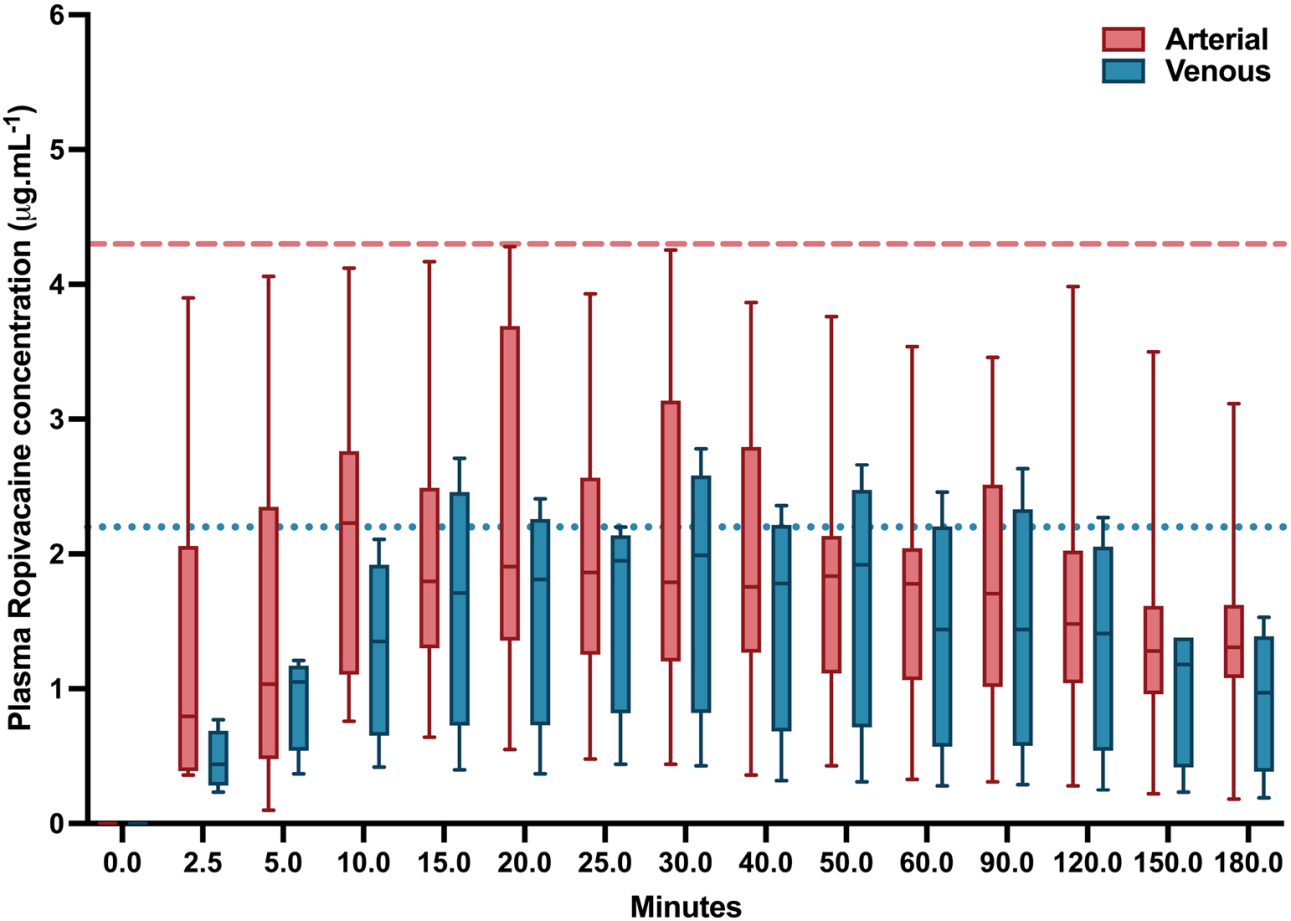
Total ropivacaine plasma arterial and venous concentrations. Data are presented as box and whisker plots with the line within the box indicating the median, the box representing the 25% and 75% percentiles, the whiskers indicating the 5% and 95% percentiles, and the single spots representing the extremes. Red dotted line = 4.3 μg/ml, blue dotted line = 2.2 μg/ml; In Knudsen study the mean tolerated arterial and venous concentration of ropivacaine was 4.3 μg/ml and 2.2 μg/ml respectively.

Ropivacaine arterial and venous plasma concentrations over time are presented in Fig. 2.

**Figure 3:**
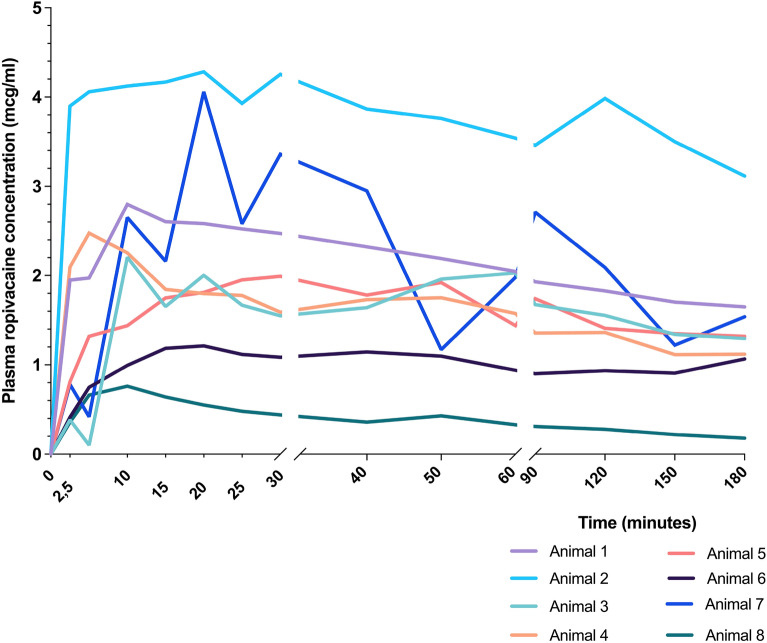
Individual arterial plasma concentration of ropivacaine.

Individual plasma concentrations of ropivacaine (µg/ml) over time are shown in Fig. 3.

The median arterial Cmax and Tmax of ropivacaine were 2.34 [1.40 to 3.74] µg/ml and 15 [10 to 20] min, respectively, and the median venous C_max_ and T_max_ were 1.99 [1.21–2.38] µg/ml and 40 min [35–40] min. The highest individual arterial and venous plasma concentrations were 4.28 µg/ml and 2.78 µg/ml attained 20 and 37 min respectively after SIFPB.

The previously described lower limit of arterial and venous plasma concentration of ropivacaine associated with toxicity (3.4 µg/ml and 0.5 µg/ml) was reached by 2 animals (25%) and 3 animals (37,5%) respectively^17^.

The electrophysiological and electrocardiographic parameters are shown in Table 2.

**Table 2:** Electrophysiological variables on the baseline and 2, 5, 10, 15, 20, 30, 40, 50, 60, 90, 120, 150 and 180 minutes after serratus intercostal fascial plane block.

Parameter	Baseline	2 min-SIFPB	5 min-SIFPB	10 min-SIFPB	15 min-SIFPB	20 min-SIFPB	30 min-SIFPB	40 min-SIFPB	50 min-SIFPB	60 min-SIFPB	90 min-SIFPB	120 min-SIFPB	150 min-SIFPB	180 min-SIFPB	P
SCL (ms)	620 ± 113	612 ± 107	618 ± 114	615 ± 119	582 ± 106	590 ± 101	596 ± 130	589 ± 128	579 ± 120	595 ± 125	589 ± 102	560 ± 94	559 ± 80	550 ± 75	0.21
PR (ms)	112 ± 7	108 ± 10	109 ± 8	112 ± 11	108 ± 15	106 ± 15	100 ± 16	106 ± 11	106 ± 11	102 ± 15	103 ± 20	102 ± 23	108 ± 14	108 ± 12	0.33
QRS (ms)	71 ± 8	75 ± 9	76 ± 10	77 ± 8	74 ± 8	75 ± 9	78 ± 11	78 ± 8	79 ± 9	76 ± 9	74 ± 11	75 ± 12	77 ± 11	77 ± 10	0.15
StQRS_400_ (ms)	93 ± 12	–	99 ± 13	99 ± 16	97 ± 11	97 ± 9	94 ± 11	96 ± 8	94 ± 9	97 ± 10	95 ± 8	97 ± 9	96 ± 7	98 ± 8	0.35
StQRS_500_ (ms)	97 ± 10	–	96 ± 10	96 ± 9	97 ± 11	95 ± 10	97 ± 7	95 ± 8	97 ± 9	95 ± 11	95 ± 11	98 ± 11	99 ± 10	98 ± 11	0.53
QT (ms)	385 ± 30	385 ± 34	389 ± 32	390 ± 38	379 ± 32	388 ± 33	382 ± 50	382 ± 61	388 ± 50	389 ± 44	401 ± 45	398 ± 40	391 ± 40	405 ± 47	0.58
QTc (ms)	489 ± 30	492 ± 25	509 ± 40	497 ± 25	496 ± 32	507 ± 24	496 ± 23	504 ± 24	513 ± 26	505 ± 20	524 ± 40	531 ± 16	522 ± 17	544 ± 44	0.01
AH (ms)	77 ± 15	78 ± 7	83 ± 9	79 ± 11	81 ± 9	79 ± 11	74 ± 19	76 ± 13	78 ± 14	81 ± 11	81 ± 9	72 ± 12	81 ± 11	75 ± 12	0.43
HV (ms)	41 ± 7	37 ± 10	36 ± 9	38 ± 6	36 ± 7	35 ± 10	39 ± 7	35 ± 11	38 ± 7	35 ± 8	35 ± 7	39 ± 11	35 ± 7	36 ± 7	0.46
Ventricular threshold (mA)	0.20 (0.20–0.37)	–	0.25 (0.20–0.37)	0.35 (0.20–0.40)	0.35 (0.22–0.5)	0.35 (0.22–0.40)	0.35 (0.22–0.4)	0.35 (0.22–0.4)	0.4 (0.3–0.4)	0.3 (0.22–0.55)	0.3 (0.22–0.5)	0.3 (0.22–0.47)	0.3 (0.22–0.5)	0.35 (0.3–0.47)	0.14
Ropivacaine* (μg/ml)	0	1.33 ± 1.24	1.46 ± 1.31	2.15 ± 1.09	2 ± 1.05	2.28 ± 1.30	2.09 ± 1.23	1.97 ± 1.07	1.78 ± 0.98	1.73 ± 0.94	1.75 ± 0.98	1.68 ± 1.08	1.41 ± 0.94	1.41 ± 0.82	0.0001

Data are shown as medians and interquartile range (IQR) for non-normally distributed data using the Shapiro–Wilk test, and as means ± standard deviation for normally distributed variables.

*SIFPB* serratus intercostal fascial plane block, *SCL* sinus cycle length, *PR* PR interval, *QRS* QRS duration, *St QRS*_*400*_ duration of stimulated QRS at a paced cycle length of 400 ms, *St QRS*_*500*_ duration of stimulated QRS at a paced cycle length of 500 ms, *QTc* corrected QT interval, *AH* atrial His interval, *HV* His-ventricular interval.

*Arterial ropivacaine concentrations.

There were no electrophysiological alterations during the study period except for the QTc interval which increased from 489 ± 30 to 544 ± 44 ms (Δ11.38 ± 6%), P = 0.01. There were no significant changes in the electrophysiological parameters evaluated at baseline and during maximal ropivacaine concentration (Table 3).

**Table 3:** Electrocardiographic and electrophysiological parameters.

Parameter	Baseline	10 min-SIFPB	P value*	20 min-SIFPB	P value^†^
SCL (ms)	620 ± 113	615 ± 119	0.67	590 ± 101	0.19
PR (ms)	112 ± 7	112 ± 11	0.94	106 ± 15	0.29
QRS (ms)	71 ± 8	77 ± 8	0.049	75 ± 9	0.21
StQRS_400_ (ms)	93 ± 12	99 ± 16	0.07	97 ± 9	0.14
StQRS_500_ (ms)	97 ± 10	96 ± 9	0.66	95 ± 10	0.36
QT (ms)	385 ± 30	390 ± 38	0.54	388 ± 33	0.57
QTc (ms)	489 ± 30	497 ± 25	0.25	507 ± 24	0.054
AH (ms)	77 ± 15	79 ± 11	0.50	79 ± 11	0.62
HV (ms)	41 ± 7	38 ± 6	0.30	35 ± 10	0.28
Ventricular threshold (mA)	0.20 (0.20–0.37)	0.35 (0.20–0.40)	0.095	0.35 (0.22–0.40)	0.15
Ropivacaine* µg/ml	0	2.15 ± 1.09	NA	2.28 ± 1.3	NA

Comparison between baseline parameters and those obtained 10 and 20 min after serratus intercostal fascial plane block coinciding with maximum arterial ropivacaine levels. N = 8.

Data are shown as medians and interquartile range (IQR) for non-normally distributed data using the Shapiro–Wilk test, and as means ± standard deviation for normally distributed variables.

*SIFPB* serratus intercostal fascial plane block, *SCL* sinus cycle length, *PR* PR interval, *QRS* QRS duration, *St QRS*_*400*_ duration of stimulated QRS at a paced cycle length of 400 ms, *St QRS*_*500*_ duration of stimulated QRS at a paced cycle length of 500 ms, *QTc* corrected QT interval, *AH* atrial His interval, *HV* His-ventricular interval.

*Arterial ropivacaine concentrations.

The hemodynamic parameters are shown in Table 4. There was a moderate decrease of 20% in mean arterial pressure values and a 23% decrease in systemic vascular resistance index (SVRI) although the values remained in physiological ranges.

**Table 4:** Hemodynamic variables on the baseline, and 10, 30, 60, 90, 150 and 180 min after serratus intercostal fascial plane block.

	Baseline	10 min post-SIFPB	30 min post-SIFPB	60 min post-SIFPB	90 min post-SIFPB	150 min post-SIFPB	180 min post-SIFPB	P value
Heart rate (b/min)	103 (84–119)	99 (81–119)	101 (94–131)	100 (84–128)	98 (93–128)	109 (91–121)	113 (97–124)	0.87
MAP (mmHg)	83 (69–91)	80 (69–89)	81 (69–84)	71 (65–83)	71 (63–86)	66 (57–91)	66 (56–85)	0.01
IC (l/min/m^2^)	3.0 (2.7–3.8)	3.1 (2.7–3.7)	3.3 (2.6–3.9)	3.1 (2.7–4.2)	3.2 (2.8–4.2)	3.5 (2.9–5.1)	3.4 (2.8–4.3)	0.8
SVRI (dynes^-1^ cm^5^ m^2^)	2044 (1533–2277)	2029 (1466–2388)	1852 (1643- 20,157)	1594 (1266–1814)	1508 (1175–1889)	1386(1148–1761)	1583 (1071–1714)	0.005
dP/dtmax (mmHg/s)	638 (596–668)	618 (505–701)	712 (610–834)	773 (585–806)	665 (555–849)	729 (539–1430)	684 (553–1210)	0.11
Ropivacaine* µg/ml	0	2.15 ± 1.09	2.09 ± 1.23	1.73 ± 0.94	1.75 ± 0.98	1.41 ± 0.94	1.41 ± 0.82	0.0001

Data are shown as medians and interquartile range (IQR).

*SIFPB* serratus intercostal fascial plane block, *SAP* systolic blood pressure, *DAP* diastolic blood pressure, *MAP* mean arterial pressure, *dP/dt*_*max*_ peak first derivative of femoral artery pressure, *SVRI* systemic vascular resistance index.

*Arterial ropivacaine concentrations.

## Discussion

The principal findings of this study were that the administration of 3 mg/kg of ropivacaine in SIFPB was associated with rapid absorption of the local anesthetic and that the concentrations achieved are similar to those described in other non-fascial chest wall blocks as PVB and intercostal nerve blocks^18–21^. We found no relevant electrophysiological toxic effects associated with the plasma concentration of ropivacaine reached after SIFP block.

To the best of our knowledge, this is the first study that has evaluated plasma concentrations of local anesthetics associated with SIFP blocks and their influence on the electrophysiological parameters. We used 3 mg/kg of ropivacaine, which is the maximum dose recommended by most authors, although this is a controversial issue as the toxicity of the local anesthetic used depends on several causes^22^. The absorption of local anesthetics and the plasma concentration achieved depends, among other factors, on the block site and its vascularization, the injection speed and the total dose administered. The chest wall is a richly vascularized region, and it is potentially expected that locoregional blocks in this area will be associated with a high peak concentration of local anesthetic. The arterial concentrations of ropivacaine observed in our study were similar to those obtained after intercostal blocks in humans, mean of 2.3 ± 0.6 µg/ml, after a ropivacaine dose of ~ 2.4 mg/kg^19^. Moreover, the peak concentration was reached with a median time of 16 min [5 to 45 min] showing a strong similarity with our data: median 15 min [10 to 20 min]. In patients undergoing thoracic PVB with a ropivacaine dose of 2 mg/kg, peak arterial concentrations were 2.47 µg/ml within 7.5 min^20^. These values are in line with ours, although we used higher doses of ropivacaine, but the time to reach the maximum concentration was shorter than in our study. This suggests that the absorption of the local anesthetic from the paravertebral space is faster compared to the serratus-intercostal interfascial space. Of note, there was significant variability in ropivacaine plasma concentrations between animals, this result is in line with the high interindividual variation in serum concentrations previously described in human’s studies^23,24^.

Bupivacaine plasma concentrations were recently assessed in patients undergoing breast surgery following unilateral or bilateral pectoral nerve blocks (PECS II) using 30–60 ml respectively of 0.25% bupivacaine with 1:400,000 epinephrine. The highest bupivacaine venous concentration after unilateral PECS was 0.038 µg/ml at 20 min^25^. In contrast in our study the highest ropivacaine venous concentration was 2.78 µg/ml attained 37 min after SIFP block. The anatomical differences of both blocks, the dose administered with and without vasoconstrictor and the study protocol in an animal vs. human model justify the discrepancies in the concentrations achieved.

Arterial plasma concentrations of ropivacaine observed in our study were higher than venous plasma concentrations, showing a large arteriovenous gradient described by other authors^17,20^. Arterial concentration is closer to that which can reach target organs such as the central nervous system (CNS) and heart, thus providing more accurate information compared to venous concentrations from a toxicological point of view^17,26^.

In healthy volunteers the arterial concentrations of ropivacaine associated with neurological symptoms were 3.4–5.3 µg/ml^17^. There is a controversial debate about the plasma concentration of ropivacaine that are considered toxic. Local anesthetic toxicity is considered to be a multifactorial phenomenon, and CNS stimulation produces an initial cardiovascular activation, which is followed by an intense depression if local anesthetic doses are sufficiently high^22^.

Different studies have evaluated the systemic concentrations of local anesthetics in abdominal fascial plane blocks. A recent review showed that, although a limited number of patients exceeded the plasma concentration considered toxic, clinical manifestations were minimal. However, most fascial plane regional blocks were performed with the patient under general anaesthesia, which precludes assessment of neurological symptoms^27^.

There are limited studies that have evaluated the effects on cardiac electrophysiology of local anesthetics at the usual plasma concentrations associated with regional blocks^28^. The administration of 200 mg of ropivacaine (~ 2.66 mg/kg) in patients undergoing interscalene brachial plexus block was associated with peak venous concentrations of ~ 2.4 µg/ml which was not followed by any electrocardiographic changes on Holter monitoring until 6 h after the block. Low doses of ropivacaine administered in healthy volunteers until the onset of neurological intoxication symptoms were associated with minimal electrocardiographic alterations, mainly an increase in QRS duration. In this study, the arterial concentrations of ropivacaine that were associated with neurological symptoms were (3.4 to 5.3 µg/ml)^17^. Our results showed that these concentrations were reached by 2 of the animals tested. However, there were no electrophysiological changes associated with peak ropivacaine concentrations compared with baseline parameters (Table 3), except for a moderate increase in the QTc interval. The QTc interval prolongation observed was significant from minute 50 to the end of the study, with a maximum increase of 55 ms at 180 min. In addition to Na + channels, ropivacaine blocks K + channels, Ca + 2 channels and HERG channels, although the action on these channels seems to be related to very high concentrations of the drug, much higher than those obtained in our study^29^. Concerning interspecies differences, it should be noted that the evaluation of potential QT-prolonging drugs is performed in animal models, and the pig is one of the useful animal species for evaluating electrocardiograms in safety pharmacology studies^30^. In this regard, it is particularly relevant to highlight the effects of sevoflurane on QTc interval prolongation shown in other studies^31,32^. Our procedure, with a duration of approximately three hours, implies a prolonged exposure to sevoflurane that may justify the increase in the QTc interval observed.

Intracardiac recordings and ventricular pacing performed in our experimental model have allowed us to assess electrophysiological events in detail, which cannot be acquired with conventional electrocardiographic monitoring. The assessment of the cardiotoxic effect of sodium channel blockade induced by local anesthetics is most sensitive when studying the effects on conduction after a short period of ventricular pacing at high rates^33^. Rate-dependent conduction slowing occurs when the sodium channel blockade developed in response to the local anesthetic has insufficient time to fully dissipate during diastole because of elevated heart rates. This unmasks a phenomenon that may be hidden in sinus rhythm by observing a large increase in QRS interval duration at high pacing rates^33^. In the previously mentioned study in healthy volunteers, transesophageal atrial stimulation at 100–120 bpm was associated with a significant (8.5%) increase in QRS width^17^. In contrast, plasma concentrations of ropivacaine after SIFPB did not affect ventricular conduction even at pacing rates of 150 bpm in our study.

Moderate hypotension was observed from baseline, before SIFPB, to the final post-SIFPB period. This slight decrease in mean arterial pressure does not appear to be related to apparent ropivacaine toxicity, since ropivacaine levels in the final phases were the lowest recorded during the entire study.

The popularity of interfascial peripheral plane blocks is related to their relative ease to perform and their hypothetically low risk profile.

One of the strengths of our study was the use of ropivacaine as a local anesthetic, which according to recent studies is one of the most commonly used local anesthetics in fascial blocks^34^.

### Limitations

Animal models allow replication of controlled clinical conditions to improve our understanding of the pharmacological and toxicological effects associated with a given regional anesthesia technique. Pigs are physiologically and anatomically close to humans and share drug metabolic pathways quite similarly to humans. Therefore, the pig is considered a very suitable model and possibly even better than many other large mammals for pharmacological research and toxicology^35^.

However, interspecies differences that limit the results obtained in our study cannot be excluded.

On the other hand, both the peak plasma concentration and the time to reach this concentration are comparable to results described in patients undergoing chest wall blocks. However, although the plasma concentrations associated with this fascial block can be inferred from the information obtained in this study, we must be cautious in extrapolating the results to what would happen in humans.

Another limitation is that there is no information from studies that have evaluated plasma concentrations of local anesthetics associated with SIFPB in humans that would allow us to make comparisons with our findings.

## Conclusions

In conclusion, the plasma concentrations of ropivacaine associated with a SIFPB in our porcine model using the maximum recommended dose of 3 mg/kg, have reached potentially toxic concentrations, considering the classic neurological toxicity margin described for ropivacaine in humans. However, they have not been associated with adverse electrophysiological or hemodynamic effects. The absorption pattern of local anesthetics in the SIFPB is similar to that of the intercostal and paravertebral blocks, both of which are also chest wall blocks.

## Data Availability

The datasets used and/or analyzed during the current study available from the corresponding author on reasonable request.
